# Classification of Rank-One Submanifolds

**DOI:** 10.1007/s00025-023-01982-8

**Published:** 2023-08-19

**Authors:** Matteo Raffaelli

**Affiliations:** Institute of Discrete Mathematics and Geometry, TU Wien, Wiedner Hauptstraße 8-10/104, 1040 Vienna, Austria

**Keywords:** Flat metric, constant nullity, ruled submanifold, striction submanifold, Primary 53A07, Secondary 53B25, 53C40, 53C42

## Abstract

We study ruled submanifolds of Euclidean space. First, to each (parametrized) ruled submanifold $\sigma$, we associate an integer-valued function, called *degree*, measuring the extent to which $\sigma$ fails to be cylindrical. In particular, we show that if the degree is constant and equal to *d*, then the singularities of $\sigma$ can only occur along an $(m-d)$-dimensional “striction” submanifold. This result allows us to extend the standard classification of developable surfaces in ${\mathbb {R}}^{3}$ to the whole family of flat and ruled submanifolds without planar points, also known as *rank-one*: an open and dense subset of every rank-one submanifold is the union of *cylindrical*, *conical*, and *tangent* regions.

## Introduction and Main Result

Developable surfaces in ${\mathbb {R}}^{3}$ are classical objects in differential geometry, enjoying a rich collection of properties. For instance, they have zero Gaussian curvature; they are envelopes of one-parameter families of planes; they are ruled, with tangent plane stable along the rulings.

Developable surfaces started to be studied in depth during the eighteenth century (even before the term “differential geometry” was coined [14]), when Leonhard Euler and Gaspard Monge got interested in their properties and classification. Their efforts led to the following, nowadays well-known, theorem.

### Theorem 1.1

([13, section 3.24]). An open and dense subset of every developable surface is a union of planar regions, cones, cylinders, and tangent surfaces of space curves.

In arbitrary dimension and codimension, a broad generalization of the notion of developable surface (without planar points) is that of *rank-one submanifold*. Let *M* be an *m*-dimensional submanifold of ${\mathbb {R}}^{m+n}$. We say that *M* is *rank-one* if the rank of the Gauss map $M \rightarrow G(m, {\mathbb {R}}^{m+n})$ is constant and equal to one; equivalently, if *M* is both flat and $(m-1)$-ruled.

Rank-one—and, more generally, constant-rank—submanifolds received a great deal of attention during the 1960s [19, p. ix], yet they continue to be the subject of intensive study; see, e.g., [3, 5, 11, 16, 18, 22].

It is therefore natural to ask whether a statement similar to that of Theorem 1.1 might hold for rank-one submanifolds [17]. In this paper, we answer such question affirmatively, by showing that there are only three types of rank-one submanifolds: *cylindrical*, *conical*, and *tangent*.

### Definition 1.2

(Cylinder). Given a smooth unit-speed curve $\gamma :I \rightarrow {\mathbb {R}}^{m+n}$ and an $(m-1)$-tuple of orthonormal vectors $X_{1}, \dotsc , X_{m-1}$, the map

$$\begin{aligned} I \times {\mathbb {R}}^{m-1}&\rightarrow {\mathbb {R}}^{m+n}\\ (t, u^{1}, \dotsc , u^{m-1})&\mapsto \gamma (t) + u^{1}X_{1} + \cdots + u^{m-1} X_{m-1} \end{aligned}$$

is called a *cylindrical submanifold* or, more simply, a *cylinder*.

### Definition 1.3

(Cone). Let $U \subset {\mathbb {R}}^{m-1}$, and let $\psi :U \rightarrow {\mathbb {R}}^{m+n}$ be a smooth map whose differential has rank $m-2$ everywhere. Given a smooth unit vector field $X_{\psi }$ along $\psi$ such that

$$\begin{aligned} X_{\psi } \notin {{\,\textrm{span}\,}}\left( \dfrac{\partial \psi }{\partial u^{1}}, \dotsc , \dfrac{\partial \psi }{\partial u^{m-1}}\right) , \end{aligned}$$

the map

$$\begin{aligned} U \times {\mathbb {R}}&\rightarrow {\mathbb {R}}^{m+n}\\ (u^{1}, \dotsc , u^{m})&\mapsto \psi (u^{1}, \dotsc , u^{m-1}) + u^{m}X_{\psi }(u^{1}, \dotsc , u^{m-1}) \end{aligned}$$

is called a *conical submanifold* or, more simply, a *cone*.

### Definition 1.4

(Tangent submanifold). Let $\xi :U \rightarrow {\mathbb {R}}^{m+n}$ be an $(m-1)$-dimensional *regular* submanifold of ${\mathbb {R}}^{m+n}$. Given a smooth unit vector field $X_{\xi }$ along $\xi$ such that

$$\begin{aligned} \dfrac{\partial \xi }{\partial u^{1}} \wedge \cdots \wedge \dfrac{\partial \xi }{\partial u^{m-1}} \wedge X_{\xi } = 0, \end{aligned}$$

the map

$$\begin{aligned} U \times {\mathbb {R}}&\rightarrow {\mathbb {R}}^{m+n}\\ (u^{1}, \dotsc , u^{m})&\mapsto \xi (u^{1}, \dotsc , u^{m-1}) + u^{m}X_{\xi }(u^{1}, \dotsc , u^{m-1}) \end{aligned}$$

is called a *tangent submanifold of* $\xi$.

### Theorem 1.5

An open and dense subset of every rank-one submanifold is a union of cylindrical, conical, and tangent submanifolds. Conversely, any cylindrical and any singular ruled submanifold of degree one, as defined in Sect. 2, is rank-one.

We emphasize that Theorem 1.5 does not involve any assumption on the codimension of *M*. On the other hand, it is worth noting that when *M* is rank-one, the first normal space (span of the image of the second fundamental form) is everywhere of dimension one; hence a rank-one submanifold is necessarily the image of an isometric composition [6], as explained by the following theorem.

### Theorem 1.6

([7, 10]). Suppose that *M* is rank-one. If the first normal bundle is parallel in the normal connection, then *M* is contained in an $(m+1)$-dimensional totally geodesic submanifold of ${\mathbb {R}}^{m+n}$. On the other hand, if the first normal bundle is not parallel, then *M* is contained in a rank-one hypersurface of ${\mathbb {R}}^{m+n}$ as a totally geodesic submanifold.

Recall that rank-one hypersurfaces can be locally described in terms of the Gauss parametrization; see [4].

The rest of the paper, which is devoted to proving Theorem 1.5, is organized as follows. After discussing some background material, in Sect. 3 we generalize the classic theory of noncylindrical ruled surfaces in ${\mathbb {R}}^{3}$ to arbitrary dimension and codimension; in particular, we verify that, under some reasonable assumption, the singularities of a ruled submanifold always concentrate along a lower dimensional *striction submanifold*. In Sect. 4 we then specialize our theory to the rank-one case and show that a noncylindrical rank-one submanifold is necessarily singular. Finally, by applying the results obtained earlier, in Sect. 5 we prove Theorem 1.5.

The classification problem for rank-one submanifold was also studied by Ushakov in his Ph.D. thesis [20]. The author came up with a classification theorem [20, Theorem 2] that looks quite similar to ours (although the techniques used in the proof do not). Unfortunately we were not able to fully understand such result and therefore cannot provide a comparison with Theorem 1.5.

## Preliminaries

In this section we discuss some preliminaries. Standard references for submanifolds are [1, 8] and [15, Chapter 8].

### Distribution Along Curves

Let $\gamma :I \rightarrow {\mathbb {R}}^{m+n}$ be a smooth regular curve. Without loss of generality, we may assume $\gamma$ to be unit-speed. A *distribution of rank* *r* *along* $\gamma$ is a rank-*r* subbundle of the ambient tangent bundle $T{\mathbb {R}}^{m+n} |_{\gamma }$ over $\gamma$. Recall that

$$\begin{aligned} T{\mathbb {R}}^{m+n}|_{\gamma } \,= \bigsqcup _{t \,\in \, I} T_{\gamma (t)}{\mathbb {R}}^{m+n}. \end{aligned}$$

Let ${\mathcal {D}}$ be a distribution of rank *r* along $\gamma$, and let ${\mathcal {D}}^{\perp }$ be the distribution of rank $m+n-r$ along $\gamma$ whose fiber at *t* is the orthogonal complement of ${\mathcal {D}}_{t}$ in ${\mathbb {R}}^{m+n}$. For each $t \in I$, define a map $\rho _{t} :{\mathcal {D}}_{t} \rightarrow {\mathcal {D}}^{\perp }_{t}$ by

$$\begin{aligned} v \mapsto \pi ^{\perp }\frac{\mathop {}\!dv}{\mathop {}\!dt}(t), \end{aligned}$$

where *v* is extended arbitrarily to a smooth local section of ${\mathcal {D}}$, and where $\pi ^{\perp }$ denotes orthogonal projection onto ${\mathcal {D}}^{\perp }$.

#### Definition 2.1

The rank of $\rho _{t}$ is called the *degree* of the distribution ${\mathcal {D}}$ at *t*. We say that ${\mathcal {D}}$ is of *degree* *d* if $d_{t} {:}{=}{{\,\textrm{rank}\,}}\rho _{t} = d$ for all $t \in I$.

#### Remark 2.2

Note that $$d_{t} \le \min (r, m+n-r)$$.

Now, let $(E_{1}, \dotsc , E_{r})$ be a frame for ${\mathcal {D}}$, i.e., an *r*-tuple of smooth vector fields along $\gamma$ forming a basis of ${\mathcal {D}}_{t}$ for all $t \in I$. In particular, suppose that $(E_{1}, \dotsc , E_{r})$ is parallel with respect to the induced connection on ${\mathcal {D}}$, so that $\pi ^{\top }(\dot{E}_{1}) = \cdots = \pi ^{\top }(\dot{E}_{r}) = 0$; here $\pi ^{\top }$ is the orthogonal projection onto ${\mathcal {D}}$. Then

$$\begin{aligned} {{\,\textrm{rank}\,}}\rho _{t} = \dim {{\,\textrm{span}\,}}( \dot{E}_{1}(t), \dotsc , \dot{E}_{r}(t)). \end{aligned}$$

### Ruled Submanifolds

A *(parametrized) submanifold of* ${\mathbb {R}}^{m+n}$ is a smooth map $V \rightarrow {\mathbb {R}}^{m+n}$, where *V* is a subset of ${\mathbb {R}}^{m}$. A point where the differential is injective is called *regular*; otherwise it is *singular*. A submanifold is itself called *regular* if all its points are regular, and *singular* otherwise.

#### Remark 2.3

A submanifold, even when regular, may have self-intersections in its image.

#### Definition 2.4

Given a smooth unit-speed curve $\gamma :I \rightarrow {\mathbb {R}}^{m+n}$ and a smooth orthonormal frame $(X_{j})_{j=1}^{m-1}$ along $\gamma$, the submanifold

$$\begin{aligned} \sigma :I \times {\mathbb {R}}^{m-1}&\rightarrow {\mathbb {R}}^{m+n}\\ (t, u^{1}, \dotsc , u^{m-1})&\mapsto \gamma (t) + u^{1} X_{1}(t) + \cdots + u^{m-1} X_{m-1}(t) \end{aligned}$$

is called a *ruled submanifold* of ${\mathbb {R}}^{m+n}$. We say that $\gamma$ is a *directrix* of $\sigma$. Moreover, for any fixed *t*, the $(m-1)$-dimensional affine subspace of ${\mathbb {R}}^{m+n}$ spanned by $X_{1}(t), \dotsc , X_{m-1}(t)$ is called a *ruling of* $\sigma$.

The simplest examples of ruled submanifolds are the cylinders (Definition 1.2). A cylinder is a ruled submanifold whose rulings are all parallel (in the usual Euclidean sense). On the other hand, a ruled submanifold is said to be *noncylindrical* if the degree of the ruling distribution ${{\,\textrm{span}\,}}(X_{j})_{j = 1}^{m-1}$ is nonzero for all $t \in I$. Similarly, we speak of ruled submanifolds of *degree* *d* if the degree of the ruling distribution is constant and equal to *d*.

Recall that the first normal space of a Riemannian submanifold at a point *p* is the linear subspace of the normal space spanned by the image of the second fundamental form at *p*. For a ruled submanifold, the degree of the ruling distribution and the dimension of the first normal space are closely related, as the next lemma shows.

#### Lemma 2.5

Let $\sigma$ be a ruled submanifold. If $\sigma$ is of degree *d*, then, at any point $p \in \sigma$, the first normal space $N_{p}^{1}\sigma$ satisfies

$$\begin{aligned} d-1 \le \dim N_{p}^{1}\sigma \le d+1. \end{aligned}$$

#### Proof

Let $X_{0} = \dot{\gamma }$, and let $II$ be the second fundamental form of $\sigma$. Since $II$ vanishes on each ruling, the first normal space is spanned by

$$\begin{aligned} II (x_{0}, x_{0}), \dotsc , II (x_{0}, x_{m-1}); \end{aligned}$$

here $x_{0} = X_{0} |_{p}$, $x_{1} = X_{1} |_{p}$, etc. Recall that

$$\begin{aligned} II (X_{0}, X_{j}) = \pi ^{\perp }_{\sigma }\dot{X}_{j} = \pi ^{\perp }_{\sigma }\rho (X_{j}), \end{aligned}$$

where $\pi ^{\perp }_{\sigma }$ denotes orthogonal projection onto $N\sigma$.

Assume that $\sigma$ is of degree *d*. Since

$$\begin{aligned} {{\,\textrm{span}\,}}\left( \rho _{t}(x_{1}), \dotsc , \rho _{t}(x_{m-1})\right) \subset N_{p}\sigma \oplus \left( T_{p}M \cap \left( {{\,\textrm{span}\,}}(x_{j})_{j = 1}^{m-1}\right) ^{\perp }\right) , \end{aligned}$$

it follows that

$$\begin{aligned} d-1 \le \dim {{\,\textrm{span}\,}}\left( II (x_{0}, x_{1}), \dotsc , II (x_{0}, x_{m-1})\right) \le d, \end{aligned}$$

from which one concludes that

$$\begin{aligned} d-1 \le \dim {{\,\textrm{span}\,}}\left( II (x_{0}, x_{0}), \dotsc , II (x_{0}, x_{m-1})\right) \le d+1. \end{aligned}$$

$$\square $$

## The Striction Submanifold

The purpose of this section is to extend the notion of line of striction of a ruled surface in ${\mathbb {R}}^{3}$ to arbitrary dimension and codimension; see, e.g., [9, section 3-5] for the classical theory.

To begin with, let us assume that $\sigma$ is a ruled submanifold. The classical line of striction is defined under the assumption that the surface $(t,u) \mapsto \gamma (t) + u X(t)$ is noncylindrical, i.e., that $\dot{X}$ never vanishes. Here we shall require that, for all $t \in I$,

$$\begin{aligned} {\left\{ \begin{array}{ll} \dim {{\,\textrm{span}\,}}( \rho _{t}X_{1}(t), \dotsc , \rho _{t}X_{m-1}(t)) = d > 0,\\ \dim {{\,\textrm{span}\,}}(\rho _{t}X_{m -d}(t), \dotsc , \rho _{t}X_{m-1}(t)) = d; \end{array}\right. } \end{aligned}$$

in other words, that both distributions ${\mathcal {D}} ={{\,\textrm{span}\,}}(X_{j})_{j=1}^{m-1}$ and ${{\,\textrm{span}\,}}(X_{h})_{h=m-d}^{m-1}$ are of degree $d >0$. Note that, by Remark 2.2, we have the inequality

$$\begin{aligned} 0 < d \le \min (m-1, n+1). \end{aligned}$$

In this setting, we are going to search for an $(m-d)$-dimensional submanifold $\beta = \beta (t, u^{1}, \dotsc , u^{m-d-1})$, lying in the image of $\sigma$ as a graph over $I \times {\mathbb {R}}^{m-d-1}$, such that $\langle \partial \beta / \partial t, \rho X_{h} \rangle = 0$ for all $h = m-d, \dotsc , m-1$; such submanifold is called the *striction submanifold of* $\sigma$. Note that $\beta$ is contained in the image of $\sigma$ as a graph over $I \times {\mathbb {R}}^{m-d-1}$ if and only if there are smooth functions $u^{m-d}, \dotsc , u^{m-1}$ such that

$$\begin{aligned} \beta (t, u^{1}, \dotsc , u^{m-d-1})&= \gamma (t) + u^{1} X_{1}(t) + \cdots + u^{m-d-1} X_{m-d-1}(t)\\&\quad + u^{m-d}(t, u^{1}, \dotsc , u^{m-d-1}) X_{m-d}(t) + \cdots \\&\quad + u^{m-1}(t, u^{1}, \dotsc , u^{m-d-1}) X_{m-1}(t). \end{aligned}$$

We thus compute

$$\begin{aligned} \frac{\partial \beta }{\partial t} = \dot{\beta }&= \dot{\gamma } + u^{1} \dot{X}_{1} + \cdots + u^{m-d-1} \dot{X}_{m-d-1}\\&\quad + \dot{u}^{m-d} X_{m-d} + u^{m-d} \dot{X}_{m-d} + \cdots \\&\quad + \dot{u}^{m-1} X_{m-1} + u^{m-1} \dot{X}_{m-1}, \end{aligned}$$

which implies that

$$\begin{aligned} \langle \dot{\beta }, \rho X_{h} \rangle = \langle \dot{\gamma }, \rho X_{h} \rangle + u^{1} \langle \dot{X}_{1}, \rho X_{h} \rangle + \cdots + u^{m-1} \langle \dot{X}_{m-1}, \rho X_{h} \rangle . \end{aligned}$$

Letting

$$\begin{aligned} A = \begin{pmatrix} \langle \dot{X}_{m-d}, \rho X_{m-d} \rangle &{} \dots &{} \langle \dot{X}_{m-1}, \rho X_{m-d} \rangle \\ \vdots &{} \ddots &{} \vdots \\ \langle \dot{X}_{m-d}, \rho X_{m-1} \rangle &{} \dots &{} \langle \dot{X}_{m-1}, \rho X_{m-1} \rangle \end{pmatrix} \in {\mathbb {R}}^{d \times d} \end{aligned}$$

and

$$\begin{aligned} b = - \begin{pmatrix} \langle \dot{\gamma }, \rho X_{m-d} \rangle + u^{1} \langle \dot{X}_{1}, \rho X_{m-d} \rangle + \cdots + u^{m-d-1} \langle \dot{X}_{m-d-1}, \rho X_{m-d} \rangle \\ \vdots \\ \langle \dot{\gamma }, \rho X_{m-1} \rangle + u^{1} \langle \dot{X}_{1}, \rho X_{m-1} \rangle + \cdots + u^{m-d-1} \langle \dot{X}_{m-d-1}, \rho X_{m-1} \rangle \end{pmatrix} \in {\mathbb {R}}^{d \times 1}, \end{aligned}$$

where $A = A(t)$ and $b = b(t, u^{1}, \dotsc , u^{m-d-1})$, our problem is equivalent to the linear system of equations

1 $$\begin{aligned} A \begin{pmatrix} u^{m-d}\\ \vdots \\ u^{m-1} \end{pmatrix} = b \end{aligned}$$

in the *d* unknowns $u^{m-d}, \dotsc , u^{m-1}$. By assumption, the matrix *A* has full rank, and so $A^{-1}b$ is the unique solution.

It remains to show that the corresponding solution $\beta$ is well-defined, meaning that its image does not depend on the choice of the curve $\gamma$. To this end, suppose that, for another choice of $\gamma$, the striction submanifold is different from the one defined by $A^{-1}b$. Then there are two striction submanifolds, say $\beta _{1}$ and $\beta _{2}$. It follows that there exist two *d*-tuples $(u_{1}^{m-d}, \dotsc , u_{1}^{m-1})$, $(u_{2}^{m-d}, \dotsc , u_{2}^{m-1})$ of functions such that

$$\begin{aligned} \beta _{\ell }(t, u^{1}, \dotsc , u^{m-d-1})&= \gamma (t) + u^{1} X_{1}(t) + \cdots + u^{m-d-1} X_{m-d-1}(t)\\&\quad + u_{\ell }^{m-d}(t, u^{1}, \dotsc , u_{\ell }^{m-d-1}) X_{m-d}(t) + \cdots \\&\quad + u_{\ell }^{m-1}(t, u^{1}, \dotsc , u_{\ell }^{m-d-1}) X_{m-1}(t), \end{aligned}$$

with $\ell =1,2$. This implies that the solution of (1) is not unique, which is a contradiction.

Having verified that the striction submanifold is well-defined, we explain its significance by proving the following proposition.

### Proposition 3.1

The only singular points of $\sigma$, if any, are along its striction submanifold. In particular, $\sigma$ is singular if and only if

$$\begin{aligned} \dot{\beta }(t, u^{1}, \dotsc , u^{m-d-1}) \wedge X_{1}(t) \wedge \cdots \wedge X_{m-1}(t) = 0 \end{aligned}$$

for some $$(t, u^{1}, \dotsc , u^{m-d-1}) \in I \times {\mathbb {R}}^{m-d-1}$$.

### Proof

First of all, we express $\sigma$ in terms of the striction submanifold:

$$\begin{aligned} \sigma (t, u^{1}, \dotsc , u^{m-1}) = \beta (t, u^{1}, \dotsc , u^{m-d-1}) + u^{m-d}X_{m-d}(t) + \cdots + u^{m-1}X_{m-1}(t). \end{aligned}$$

To find the singularities of $\sigma$, we compute its partial derivatives, take their wedge product *Z*, and then set it equal to zero.

The partial derivatives of $\sigma$ with respect to *t* and $u^{j}$ are

$$\begin{aligned} \frac{\partial \sigma }{\partial t} = \dot{\beta } + u^{m-d}\dot{X}_{m-d} + \cdots + u^{m-1}\dot{X}_{m-1} \end{aligned}$$

and

$$\begin{aligned} \frac{\partial \sigma }{\partial u^{j}} = {\left\{ \begin{array}{ll} X_{j} + \dfrac{\partial u^{m-d}}{\partial u^{j}} X_{m-d} + \cdots + \dfrac{\partial u^{m-1}}{\partial u^{j}} X_{m-1} \quad &{}\text {if } j \in \{1, \dotsc , m-d-1\},\\ X_{j} \quad &{}\text {if } j \in \{m-d, \dotsc , m-1\}, \end{array}\right. } \end{aligned}$$

respectively. Hence, computing their wedge product gives

$$\begin{aligned} Z&= \left( \dot{\beta } + u^{m-d}\dot{X}_{m-d} + \cdots + u^{m-1}\dot{X}_{m-1} \right) \\&\quad \wedge \left( X_{1} + \dfrac{\partial u^{m-d}}{\partial u^{1}} X_{m-d} + \cdots + \dfrac{\partial u^{m-1}}{\partial u^{1}} X_{m-1}\right) \wedge \cdots \\&\quad \wedge \left( X_{m-d-1} + \dfrac{\partial u^{m-d}}{\partial u^{m-d-1}} X_{m-d} + \cdots + \dfrac{\partial u^{m-1}}{\partial u^{m-d-1}} X_{m-1}\right) \wedge X_{m-d} \wedge \cdots \wedge X_{m-1}\\&= \left( \dot{\beta } + u^{m-d}\dot{X}_{m-d} + \cdots + u^{m-1}\dot{X}_{m-1}\right) \wedge X_{1} \wedge \cdots \wedge X_{m-1}. \end{aligned}$$

We claim that $Z = 0$ exactly when

$$\begin{aligned} {\left\{ \begin{array}{ll} u^{m-d} = \cdots = u^{m-1} = 0,\\ \dot{\beta } \wedge X_{1} \wedge \cdots \wedge X_{m-1} = 0. \end{array}\right. } \end{aligned}$$

To verify the claim, note that $Z(t, u^{1}, \dotsc , u^{m-d-1}) = 0$ if and only if

$$\begin{aligned}&\dot{\beta }(t, u^{1}, \dotsc , u^{m-d-1}) + u^{m-d} \dot{X}_{m-d}(t) + \cdots + u^{m-1} \dot{X}_{m-1}(t)\\&\qquad = a_{1} X_{1}(t) + \cdots + a_{m-1} X_{m-1}(t) \end{aligned}$$

for some scalars $a_{1}, \dotsc , a_{m-1}$, i.e.,

2 $$\begin{aligned} \begin{aligned} \dot{\beta }(t, u^{1}, \dotsc , u^{m-d-1})&= a_{1} X_{1}(t) + \cdots + a_{m-1} X_{m-1}(t)\\&\quad - u^{m-d} \dot{X}_{m-d}(t) - \cdots - u^{m-1} \dot{X}_{m-1}(t). \end{aligned} \end{aligned}$$

On the other hand, by definition of $\beta$, we have $\langle \dot{\beta }, \rho X_{m-d}\rangle = \cdots = \langle \dot{\beta }, \rho X_{m-1}\rangle =0$. Hence (2) implies

$$\begin{aligned} {\left\{ \begin{array}{ll} u^{m-d} \langle \dot{X}_{m-d}(t), \rho _{t} X_{m-d}(t)\rangle + \cdots + u^{m-1} \langle \dot{X}_{m-1}(t), \rho _{t} X_{m-d}(t)\rangle =0,\\ \vdots \\ u^{m-d} \langle \dot{X}_{m-d}(t), \rho _{t} X_{m-1}(t)\rangle + \cdots + u^{m-1} \langle \dot{X}_{m-1}(t), \rho _{t} X_{m-1}(t)\rangle =0. \end{array}\right. } \end{aligned}$$

This defines a homogenous linear system of full rank, which has unique (trivial) solution; thus $u^{m-d} = \cdots = u^{m-1} = 0$. $\square$

### Remark 3.2

Suppose that $\rho _{t} X_{j}(t) \ne 0$. Since the vectors $\dot{\beta }(t, u^{1},\dotsc , u^{m-d-1})$, $X_{1}(t), \dotsc , X_{m-1}(t)$ are in the orthogonal complement of $\rho _{t} X_{j}(t)$, the condition

$$\begin{aligned} \dot{\beta }(t, u^{1},\dotsc , u^{m-d-1}) \wedge X_{1}(t) \wedge \cdots \wedge X_{m-1}(t) = 0 \end{aligned}$$

is equivalent to

$$\begin{aligned} \dot{X}_{j}(t) \wedge \dot{\beta }(t, u^{1},\dotsc , u^{m-d-1}) \wedge X_{1}(t) \wedge \cdots \wedge X_{m-1}(t) = 0. \end{aligned}$$

### Remark 3.3

The proof of Proposition 3.1 shows that the dimension of the image of $\mathop {}\!d\sigma$ at a singular point is $m-1$. Since, whenever $u^{m-d} = \cdots = u^{m-1} = 0$,

$$\begin{aligned} Z = \dot{\beta } \wedge \dfrac{\partial \beta }{\partial u^{1}} \wedge \cdots \wedge \dfrac{\partial \beta }{\partial u^{m-d-1}} \wedge X_{m-d} \wedge \cdots \wedge X_{m-1}, \end{aligned}$$

it follows that the image of $\mathop {}\!d\beta _{(t,u)}$ has dimension at least equal to $m-d-1$ for all $(t,u) \in I \times {\mathbb {R}}^{m-d-1}$.

## Rank-One Submanifolds

Here we study an important subclass of ruled submanifolds, called *rank-one*.

### Definition 4.1

Let $\alpha$ be an *m*-dimensional submanifold of ${\mathbb {R}}^{m+n}$. The *relative nullity index* of $\alpha$ at a regular point *q* is the dimension of the kernel of the second fundamental form of $\alpha$ at *q* [2]. We say that $\alpha$ is a *rank-one* submanifold if it is ruled, and the relative nullity index at *q* is equal to $m-1$ for all regular points $q \in \alpha$.

### Remark 4.2

The kernel of the second fundamental form of $\alpha$, also known as the *relative nullity distribution*, coincides with the kernel of the differential of the Gauss map $\alpha \rightarrow G(m, {\mathbb {R}}^{m+n})$ [8, Exercise 1.24].

### Remark 4.3

Requiring that $\alpha$ be ruled in Definition 4.1 is only done for ease of presentation, as we previously defined ruled submanifolds as foliated by *complete* leaves.

The next result characterizes rank-one submanifolds in two different ways.

### Theorem 4.4

([21, 12, Lemma 3.1]). If $\sigma$ is a ruled submanifold without planar points, then the following statements are equivalent:

1. $\sigma$ is rank-one.
2. The induced metric on $\sigma$ is flat.
3. All tangent spaces along (the regular points of) any fixed ruling can be canonically identified with the same linear subspace of ${\mathbb {R}}^{m+n}$.

According to Theorem 4.4, a ruled submanifold $\sigma$ that is free of planar points is rank-one exactly when the span of its partial derivatives is independent of $u^{1}, \dotsc , u^{m-1}$. As explained in [18, section 3], this condition is equivalent to $\dot{X}_{j}$ being tangent to $\sigma$ for all $j = 1, \dotsc , m-1$.

### Lemma 4.5

([18, Corollary 3.8]). If $\sigma :(t,u) \mapsto \gamma (t) + u^{j}X_{j}(t)$ is rank-one, then the following system of equations holds:

3 $$\begin{aligned} {\left\{ \begin{array}{ll} \dot{X}_{1} \wedge \dot{\gamma } \wedge X_{1} \wedge \cdots \wedge X_{m-1} = 0, \\ \vdots \\ \dot{X}_{m-1} \wedge \dot{\gamma } \wedge X_{1} \wedge \cdots \wedge X_{m-1} = 0. \end{array}\right. } \end{aligned}$$

Conversely, suppose that $\sigma$ has no planar point and is regular along $\gamma$. If (3) holds, then $\sigma$ is rank-one.

This lemma implies that a noncylindrical rank-one submanifold is necessarily of degree one, because (3) forces $\rho _{t} X_{j}(t)$ to lie in the orthogonal complement of ${\mathcal {D}}_{t} = {{\,\textrm{span}\,}}(X_{j}(t))_{j=1}^{m-1}$ in the tangent space of $\sigma$, which is a subspace of dimension one.

A further consequence of Lemma 4.5 is that noncylindrical rank-one submanifolds necessarily have singularities.

### Proposition 4.6

If $\sigma$ is rank-one and noncylindrical, then all points of the striction hypersurface are singular. Conversely, if $\sigma$ is a ruled submanifold of degree one that is singular at $\beta (t, u^{1}, \dotsc , u^{m-2})$ for all $t \in I$ and some $(u^{1}, \dotsc , u^{m-2}) \in {\mathbb {R}}^{m-2}$, then it is rank-one.

### Proof

Once and for all, suppose that $d = 1$. Then

4 $$\begin{aligned} \begin{aligned} \dot{\beta }(t, u^{1}, \dotsc , u^{m-2})&= \dot{\gamma }(t) + u^{1} \dot{X}_{1}(t) + \cdots + u^{m-2} \dot{X}_{m-2}(t)\\&\quad + \dot{u}^{m-1}(t, u^{1}, \dotsc , u^{m-2}) X_{m-1}(t)\\&\quad + u^{m-1}(t, u^{1}, \dotsc , u^{m-2}) \dot{X}_{m-1}(t). \end{aligned} \end{aligned}$$

Thus, when $\sigma$ is rank-one,

5 $$\begin{aligned} \dot{X}_{j}(t) \wedge \dot{\beta }(t, u^{1}, \dotsc , u^{m-2}) \wedge X_{1}(t) \wedge \cdots \wedge X_{m-1}(t) = 0, \end{aligned}$$

which, by Remark 3.2, implies

$$\begin{aligned} \dot{\beta } \wedge X_{1} \wedge \cdots \wedge X_{m-1} = 0. \end{aligned}$$

Conversely, if $\sigma$ is singular at $\beta (t, u^{1}, \dotsc , u^{m-2})$, then (5) holds. Substituting (4), we obtain

$$\begin{aligned} \dot{X}_{j}(t) \wedge \dot{\gamma }(t) \wedge X_{1}(t)\wedge \cdots \wedge X_{m-1}(t) = 0, \end{aligned}$$

as desired. $$\square $$

## Proof of Theorem 1.5

We may now finalize the proof of our main result, Theorem 1.5 in the introduction.

Suppose that $\sigma$ is a noncylindrical rank-one submanifold. Then

$$\begin{aligned} \sigma (t, u^{1}, \dotsc , u^{m-1}) = \beta (t, u^{1}, \dotsc , u^{m-2}) + u^{m-1}X_{m-1}(t) \end{aligned}$$

and, by Proposition 4.6, all points of the striction hypersurface $\beta$ are singular points of $\sigma$; besides, by Remark 3.3, $\dim d\sigma = m-1$ along $\beta$.

We shall distinguish two cases:

1. The striction hypersurface $\beta$ is regular. Then $\sigma$ is a tangent submanifold of $\beta$.
2. The striction hypersurface is singular everywhere, i.e., $\dim d\beta _{(t,u)} = m-2$ for all $(t,u) \in I \times {\mathbb {R}}^{m-2}$. Then $\sigma$ is a conical submanifold.

Conversely, if $\sigma$ is a conical or a tangent submanifold of degree one, then it is ruled and singular. Hence, by Proposition 4.6, it is rank-one.

## Data Availability

Data sharing not applicable to this article as no datasets were generated or analyzed during the current study.
